# Development of an Efficient Strategy to Improve Extracellular Polysaccharide Production of *Ganoderma lucidum* Using L-Phenylalanine as an Enhancer

**DOI:** 10.3389/fmicb.2019.02306

**Published:** 2019-10-15

**Authors:** Zhongbao Ma, Mengmeng Xu, Qiong Wang, Feng Wang, Huihua Zheng, Zhenghua Gu, Youran Li, Guiyang Shi, Zhongyang Ding

**Affiliations:** ^1^Key Laboratory of Carbohydrate Chemistry and Biotechnology, Ministry of Education, School of Biotechnology, Jiangnan University, Wuxi, China; ^2^National Engineering Laboratory for Cereal Fermentation Technology, Jiangnan University, Wuxi, China; ^3^School of Food and Biological Engineering, Jiangsu University, Zhenjiang, China; ^4^Jiangsu Alphay Biological Technology Co., Ltd., Nantong, China

**Keywords:** *Ganoderma lucidum*, extracellular polysaccharide, L-phenylalanine, cell wall, efficient strategy

## Abstract

*Ganoderma lucidum* has been a well-known species of basidiomycetes for a long time, and has been widely applied in the fields of food and medicine. Based on the simulation results of model *i*ZBM1060 in our previous research, the effect of L-phenylalanine on *G. lucidum* extracellular polysaccharides (EPSs) was investigated in this study. EPS production reached 0.91 g/L at 0.4 g/L L-phenylalanine after a 24 h culture, which was 62.5% higher than that of control (0.56 g/L). Transcriptome and genome analysis showed that L-phenylalanine deaminase and benzoate 4-hydroxylase (related to L-phenylalanine metabolism) were significantly up-regulated, while the cell wall mannoprotein gene was down-regulated. Transmission electronic microscopy (TEM) and atomic force microscopy results showed that the cell wall thickness decreased by 58.58%, and cell wall porosity increased in cells treated with L-phenylalanine, which probably contribute to the increasing EPS production. This study provides an efficient strategy for fungal polysaccharide production with high output and low cost.

## Introduction

*Ganoderma lucidum* is an important variety of mushroom that possesses a wide range of biological activities. *G. lucidum* polysaccharide, as one of the main bioactive substances with a complex structure, has been studied for its anticancer and immunomodulatory activities (Liu et al., 2010; Ferreira et al., 2015; Xiang et al., 2018; Zhen et al., 2018). In the latest studies, liquid fermentation technology of *G. lucidum* prospered due to its ability to enhance mycelial biomass and produce more bioactive compounds with high efficiency (Zhou et al., 2014; Wei et al., 2016, 2018).

Great efforts have been made to improve the polysaccharide production of *G. lucidum*. In our previous research, the *G. lucidum* genome metabolic model *i*ZBM1060 was reconstructed. The simulation and predictions based on this model indicated that L-phenylalanine had a significant promoting effect on the production of EPSs (Ma et al., 2018). L-phenylalanine is an aromatic amino acid with physiological activities; it is one of the essential amino acids that cannot be naturally synthesized by the human body and animals and is widely applied in the food and medicine industries (Bongaerts et al., 2001). To our best knowledge, few studies have investigated the impacts of L-phenylalanine on the cultivation of edible and medicinal fungi.

In this study, an efficient strategy for EPS production with high output was established by adding L-phenylalanine to the culture of *G. lucidum*. The possible metabolic pathway of L-phenylalanine in *G. lucidum* was elucidated, and the mechanism of L-phenylalanine that promotes EPS production was also investigated.

## Materials and Methods

### *G. lucidum* Strain and Culture Conditions

*Ganoderma lucidum* CGMCC5.26 was purchased from the China General Microbiological Culture Collection Center (Beijing). It was preserved on potato dextrose agar (PDA) slants at 4°C. The composition of the seed and submerged fermentation medium (g/L) is as follows: glucose (20), tryptone (5), yeast nitrogen base without amino acids (YNB) (5), and MgSO_4_⋅7H_2_O (2), KH_2_PO_4_ (4.5), with culture conditions of 30°C at 150 rpm (Ma et al., 2018).

Medium A (g/L) (Tang and Zhong, 2002) contained glucose (35), tryptone (5), yeast extract (2.5), KH_2_PO_4_⋅H_2_O (1), MgSO_4_⋅7H_2_O (0.5), and Vitamin B_1_ (0.05), with culture conditions of 30°C at 150 rpm. Medium B (g/L) (Hu et al., 2018) contained glucose (20), tryptone (2), yeast extract 2, KH_2_PO_4_ (4.6), and MgSO_4_⋅7H_2_O (0.5), with culture conditions of 30°C at 150 rpm.

The seed culture included four mycelium agar squares (3 mm × 3 mm), which were transferred into a 250 mL flask and cultured for 7 days. The fermentation culture was developed as follows. After homogenization with a high-speed tissue homogenate machine (IKA T10 basic ULTRA-TURRAX), a 3 mL of the seed culture solution was inoculated into a 500 mL flask and cultured for 7 days (Peng et al., 2015).

### Determination of the Biomass, Residual Sugar in Medium, EPS, and IPS

After the end of fermentation, mycelium was obtained by centrifugation (10,000 rpm, 10 min); it was then washed with distilled water three times and dried to a constant weight (60°C). The DW was measured by the gravimetric method.

For EPS, we added 95% (v/v) ethanol (four times) into the above supernatant, then stirred it evenly and placed it in a 4°C environment for 8 h. Crude EPSs were acquired through centrifugation (8,000 rpm for 20 min), washed with 80% (v/v) ethanol three times, and further dried to remove the residual ethanol (60°C). The phenol-sulfuric acid method was used to determine the total EPS content (Dubois et al., 1951).

For the IPS analysis (Stajic et al., 2011), 100 mg mycelia powder was suspended in 10 mL H_2_O at 100°C for 3 h. The supernatant was then collected by centrifugation (8,000 rpm for 20 min) and the supernatant volume was recorded. The extraction method of IPS in the supernatant was the same as that of EPS. The total IPS content was assayed by the phenol-sulfuric acid method.

### Ultra-Structural Analysis of *G. lucidum*

In order to detect the function of L-phenylalanine on the ultra-structure, *G. lucidum* cells were analyzed by TEM. *G. lucidum* cells were fixed in 5% glutaraldehyde (0.1 M phosphate buffer, pH 7.2) and then rinsed with 0.1 M phosphate buffer. Next, the cells were fixed again with 1% osmium acid (0.1 M phosphate buffer, pH 7.2) and rinsed with 0.1 M phosphate buffer. The cell samples were dehydrated with ethanol and embedded in Epon 812 resin. Finally, the embedded cells were sliced and then stained in a saturated uranyl acetate and aqueous lead citrate solution and placed in a TEM (HITACHI H-7650) to observe theie ultra-structures (Armando et al., 2012).

The thickness of the cell wall was measured near the equatorial cut surfaces using the Image J software. To guarantee the accuracy of the analysis, we only selected the cells of approximately the same size, and the cells were cut longitudinally (Tavernier et al., 2018).

To observe the cell surface, the mycelium was washed three times with sterile water and then homogenized. The sample was placed on the silicon wafer (0.5 cm × 0.5 cm). The silicon wafer was mounted on a steel disc using two-sided tape. The steel disc was then magnetically attached to the piezoscanner. The microstructures of the samples were observed by an AFM (Bruker MultiMode 8). The images were then analyzed and processed using NanoScope Analysis (Kim et al., 2007).

### RNA Sequencing and Gene Expression Analysis

The total RNA from the mycelial samples with and without the addition of L-phenylalanine was extracted using an RNeasy Mini Kit (Qiagen, Hilden, Germany) according to the manufacturer’s protocol. Then, RNA degradation and contamination, purity, concentration, and integrity were measured using 1% agarose gels, a NanoPhotometer spectrophotometer (IMPLEN, Westlake Village, CA, United States), a Qubit RNA Assay Kit in Qubit 2.0 Fluorometer (Life Technologies, Carlsbad, CA, United States), and an RNA Nano 6000 Assay Kit of the Agilent Bioanalyzer 2100 system (Agilent Technologies, Santa Clara, CA, United States), respectively (Guan et al., 2018; Liu et al., 2018).

A total of 1.5 μg RNA per sample was used to prepare the sequencing libraries using the NEBNext Ultra^TM^ RNA Library Prep Kit for Illumina (NEB, United States) following the manufacturer’s recommendations. The final PCR products were sequenced via Illumina HiSeq^TM^ 2500 (Novogene Co., Ltd., Beijing, China). Before proceeding with the analysis, two steps (removing reads containing the adapter, reads containing ploy-N, and low quality reads and calculating Q20, Q30, GC-content, and sequence duplication level) were needed to confirm that the clean data quality was sufficient for the sequence assembly and subsequent analysis. Transcriptome assembly was accomplished based on the left.fq and right.fq using Trinity. A subsequent analysis was performed by genome mapping (based on the *G. lucidum* 5.26 genome sequence), functional gene annotation and classification (using the following databases: Nr, Nt, Pfam, KOG/COG, Swiss-Prot, KO and GO). Gene expression levels were calculated using TPM (Transcripts Per Kilobase of exon model per Million mapped reads) (Gu et al., 2017). Differentially expressed genes were identified according to the threshold of the *q*-value <0.005 (Storey, 2003) and |log_2_ (Fold change)| > 1 by using the DEGseq (2010) R package (Robinson et al., 2010).

### Real-Time Quantitative PCR Analysis of Gene Expression

The transcription levels of the GL21534-R1, GL28294-R1, and GL18553-R1 genes (which encode the enzymes of L-phenylalanine deaminase, benzoate 4-hydroxylase, and the cell wall mannoprotein, respectively) were analyzed by real-time quantitative PCR (RT-qPCR). The PCR reaction procedure was as follows. After denaturation at 95°C for 10 min, the amplification was divided into denaturation at 95°C for 15 s, annealing at 60°C for 1 min, and extension at 60°C for 15 s (Xu et al., 2010). The *rns* was used as a reference gene, as reported previously (Li et al., 2013). The expression of the related genes was calculated according to the 2^−ΔΔ*C*_t_^ method (Livak and Schmittgen, 2001). The primers used are listed in Table 1.

**TABLE 1:** Oligonucleotide primers used in this study.

**Target gene**	**Primer name**	**Sequence (5′ → 3′)**
*pal*	pal-F	GCTCATCGGCAACCCATCTA
	pal-R	CCGTTGAGGATACCGAGGTG
*bpha*	bpha-F	GGGTGTGGTACGACTGTCTG
	bpha-R	GAGTCCTTACACGCGAGGAG
*pir*	pir-F	TTCGCCAAACCGTTGCATTC
	pir-R	GGGTTTGCGGAGACATGAGA
*rns*	rns-F	GAGAAACGAAGGTTAGGGTAGG
	rns-R	CACAAGGCGGAATGGTTATTG

### Quantification of β-1,3-Glucan and Chitin

Mycelium powder was suspended in distilled water, and cell fragmentation was obtained by ultrasonication for 10 min (300 W, 30% duty cycle, Sonics 750). The degree of cell breakage was observed by microscope. The broken cells were washed by the washing solution in the following order: washing solution A [1 mM phenylmethylsulfonyl fluoride (PMSF)], washing solution B [5% (w/v) NaCl, 1 mM PMSF], washing solution C [2% (w/v) NaCl, 1 mM PMSF], washing solution D [1% (w/v) NaCl, 1 mM PMSF], and then the intracellular contaminants were removed with wash solution A (Chen et al., 2012). Each step was repeated three times, and the isolated cell wall material was lyophilized.

The β-1,3-glucan and chitin concentrations were measured using a previously described method (Fortwendel et al., 2009). The measurement of β-1,3-glucan was as follows. A 20 mg cell wall sample was suspended in a 2 mL 1 M NaOH solution and mixed, It was then incubated (52°C for 30 min). A total of 0.5 mL of supernatant was aliquoted into a new tube, and 1.85 mL of an aniline blue mix was added for incubation (52°C for 30 min). The fluorescence value was detected via fluorescence spectrophotometer (HITACHI F-7000) at 405 nm excitation and 460 nm emission. The β-1,3-glucan content was expressed as the relative fluorescence unit percentage per milligram of mycelium.

The measurement of the chitin was performed as follows. A 20 mg cell wall sample was added to 3 mL of a saturated KOH solution at 130°C for 1 h. After cooling to room temperature, the sample was added to 10 mL of an ice-cold 75% (v/v) ethanol solution, shaken to form a suspension, and then given an ice bath for 15 min. Next, after adding 0.9 mL of 13.3% (w/v) celtite545, the mixture was shaken for 5 min and centrifuged at 4°C at 5,000 rpm for 5 min. The precipitation was cleaned by 10 mL of ice-cold 40% (v/v) ethanol and 10 mL of ice-cold distilled water, respectively. The sample was prepared by centrifuging to remove the solution and adding 20 mL of distilled water, taking 0.5 mL of the mixture as sample. The standard solution (10 μg/mL glucosamine) and blank control (distilled water) were prepared. 0.5 mL of water, 5% (w/v) NaNO_2_, and 5% (w/v) KHSO_4_ were added to 0.5 mL of the sample, and then gently mixed for 15 min. The solution was then centrifuged at 10,000 rpm for 5 min at 4°C to remove precipitation. A total of 0.5 mL of 12.5% (w/v) NH_4_ sulfamate was added to the 0.5 mL sample and vigorously mixed for 5 min. Then, 0.5 mL of an 0.5% (w/v) newly prepared 3-methylbenzthiazolinone-2-hydrazone solution was added. The sample was mixed and boiled for 3 min. After cooling to room temperature, 0.5 mL of 0.83% (w/v) FeCl_3_⋅6H_2_O was added. After being maintained at room temperature for 30 min, the absorbance value was measured with a spectrophotometer at a wavelength of 650 nm. According to formula (1), the glucosamine contention decomposed by chitin can be calculated.

(1) $$X=\frac{A-A_{0}}{A_{1}-A_{0}}\times 10$$

where *X* is the concentration of glucosamine (μg/mL), *A*_1_ is the standard absorbance value, *A*_0_ is the distilled water absorbance value, *A* is the sample absorbance value, and 10 is the known glucosamine concentration (μg/mL).

### Statistical Analysis

All the experiments were triplicated. The results were indicated as the mean ± standard deviation by the SPSS 16.0 software (SPSS, Inc., Chicago, IL, United States). The data were analyzed by a one-way analysis of variance (ANOVA), and the differences between the means were determined by Tukey’s multiple-range tests. The results were regarded as statistically significant at *P* < 0.05.

## Results and Discussion

### Effect of L-Phenylalanine on the EPS Production of *G. lucidum*

**FIGURE 1:**
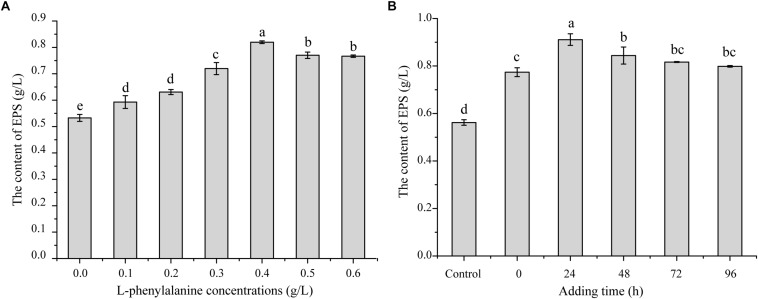
The effect of L-phenylalanine on EPS production in *G. lucidum*. **(A)** Effects of L-phenylalanine concentrations on EPS. **(B)** Effects of L-phenylalanine (0.4 g/L) addition at different stage on EPS [the different superscript letters (a, b, c, d) in each column indicate significant differences at the *P* < 0.05 level].

In the preliminary work of our laboratory, it was found that adding L-phenylalanine to a fermentation medium could improve the *G. lucidum* EPS yield based on the model *i*ZBM 1060 (Ma et al., 2018). In order to test the effect of L-phenylalanine, different concentrations of L-phenylalanine were added to the medium of *G. lucidum*. As shown in Figure 1A, L-phenylalanine at 0.4 g/L provided the maximal EPS production of 0.79 g/L, and EPS production increased by 45.49%. In order to better understand the effects of L-phenylalanine on the production of polysaccharides, 0.4 g/L L-phenylalanine was added at different time periods. The EPS yield improved from 0.56 to 0.91 g/L by adding L-phenylalanine at 24 h, leading to a 62.50% significant increase (Figure 1B). Thus, it can be seen that L-phenylalanine is very important to improve EPS synthesis efficiency. Accordingly, 0.4 g/L L-phenylalanine added at 24 h was selected for subsequent studies.

**FIGURE 2:**
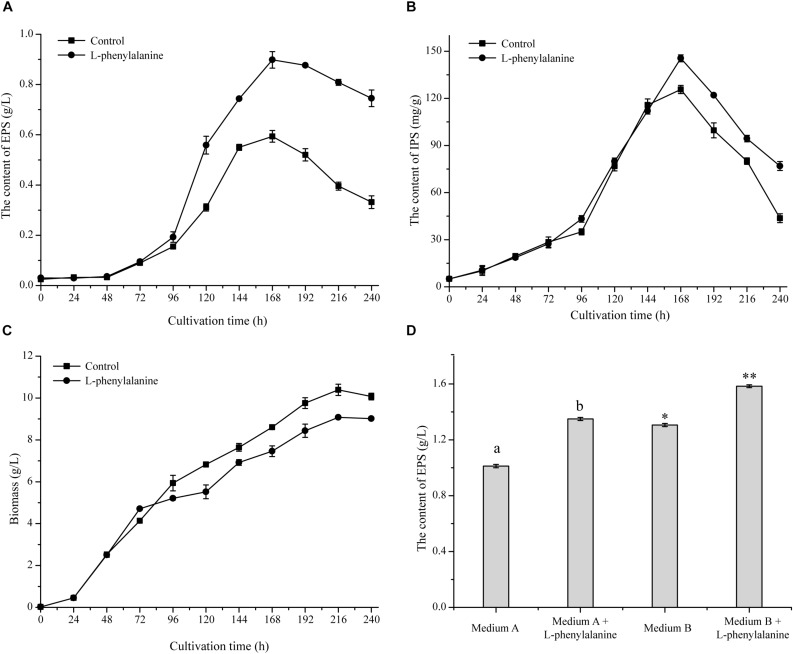
Effect of L-phenylalanine on the yield of EPS **(A)**, IPS **(B)**, and cell growth (by biomass) **(C)**. **(D)** The effect of L-phenylalanine on the yield of EPS in different culture media [the different superscript letters (a, b) and symbols (^∗^, ^∗∗^) in each column indicate significant differences at the *P* < 0.05 level].

The maximum specific EPS production rate [Q_EPS_, calculated by the formula (1/*X*) × (d*P*_EPS_/d*t*), where *X* means cell biomass, and *P*_EPS_ means EPS production] of *G. lucidum* was achieved by adding L-phenylalanine. As shown in Figure 2A, the Q_EPS_ reached 2.96 mg/g DW/h, which was a 1.71-fold increase compared to the control. The addition of L-phenylalanine also promoted the content of IPS, which increased to 145.51 mg/g DW, with a 15.79% increment (Figure 2B). The experimental results show that L-phenylalanine contributed to the improvement of Q_EPS_ and IPS concentration, thereby indicating that L-phenylalanine facilitated polysaccharide synthesis in the submerged *G. lucidum* culture. However, L-phenylalanine had a certain negative effect on the growth of *G. lucidum* and caused a 12.61% decrease in maximal biomass (Figure 2C).

Furthermore, the effects of L-phenylalanine on *G. lucidum* EPS production were tested in other reported culture media (Figure 2D). Results showed that the addition of L-phenylalanine could increase the maximum EPS yield by 1.33-fold (medium A) and 1.21-fold (medium B). Due to the wide production and lower price of L-phenylalanine, it is economically feasible to use L-phenylalanine as an efficient intensification strategy to promote the EPS production of *G. lucidum*.

Indeed, L-phenylalanine could also promote the synthesis of the intended products in other culture systems. A high concentration of L-phenylalanine led to the highest phenylalanine ammonia-lyase activities and improved colchicine production (Sivakumar et al., 2004). L-phenylalanine had a stimulative effect on the production of phenylethanoid glycosides in *Cistanche deserticola*, and maximal phenylethanoid glycoside production at 1.5 mmol/L of L-phenylalanine was 1.13-fold higher than the control (Hu et al., 2014). A certain concentration of L-phenylalanine enhanced the flavonoid production of *Inonotus baumii* (Mathur and Goswami, 2014; Li et al., 2018).

### Comparative Analysis of the *G. lucidum* 5.26 Transcriptomes

**FIGURE 3:**
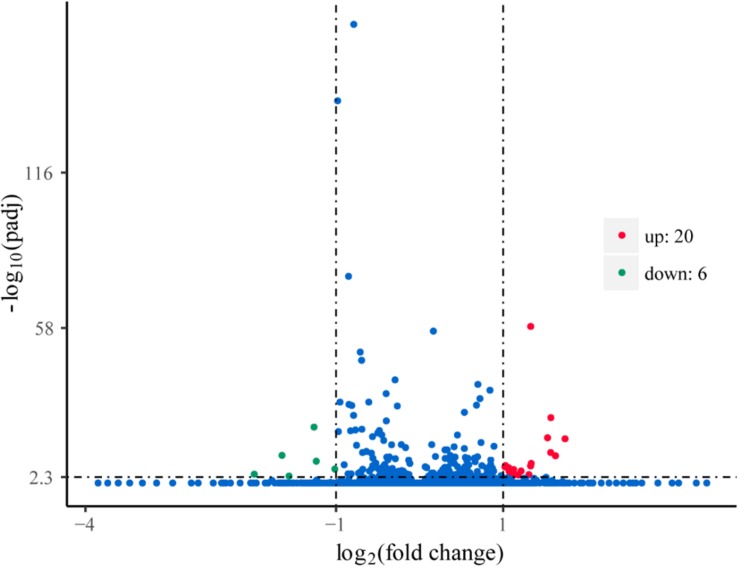
Volcano plots of gene expression. The points of the green areas represent down-regulated genes, the points of the red areas represent up-regulated genes, and other points represent genes without significant change.

To explore the genes in response to L-phenylalanine, we compared the gene expression after L-phenylalanine treatment at a global level (Supplementary Table S1). The differentially expressed genes in *G. lucidum* under different culture conditions are displayed in Figure 3, a total of 20 up-regulated and 6 down-regulated genes. The 26 genes were annotated through the KAAS (Moriya et al., 2007) and UniProt (2010) databases. In the annotation results, two key enzymes related to L-phenylalanine metabolism were found to be up-regulated, and the cell wall synthesis related gene was found to be down-regulated.

Based on RT-qPCR analysis, the expression levels of two key enzymes related to L-phenylalanine metabolism, L-phenylalanine deaminase (*pal*, EC: 4.3.1.24, GL21534-R1), and benzoate 4-hydroxylase (*bpha*, EC: 1.14.14.92, GL28294-R1) were up-regulated by 264.27 and 125.81%, respectively. L-phenylalanine deaminase catalyzes the deamination of L-phenylalanine and then produces *trans*-cinnamic acid. Benzoate 4-hydroxylase catalyzes the benzoate to form 4-hydroxybenzoate.

**FIGURE 4:**
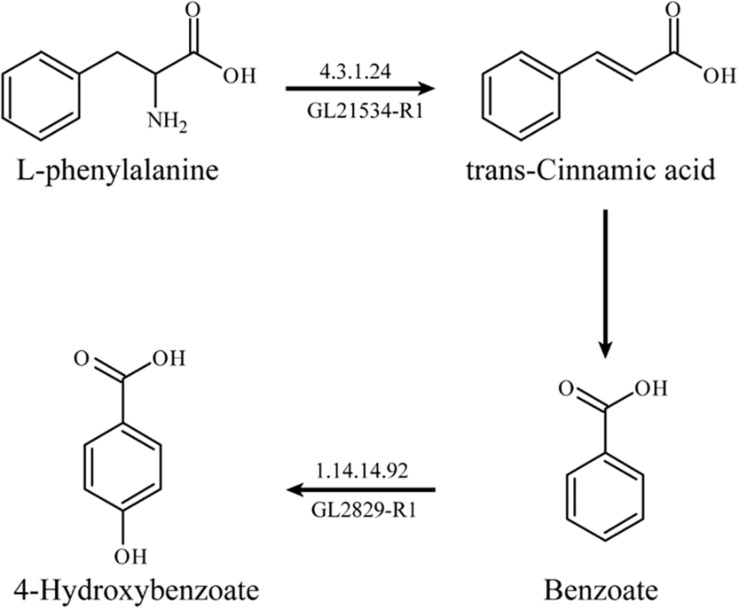
The metabolic pathway of L-phenylalanine in *G. lucidum.*

The possible metabolic pathway of L-phenylalanine in *G. lucidum* can be determined according to the results of the transcriptome analysis and genome annotation. L-phenylalanine deaminase catalyzed the deamination of L-phenylalanine to generate *trans*-cinnamic acid and benzoate accordingly, and then benzoate was catalyzed by benzoate 4-hydroxylase to form 4-hydroxybenzoate (Figure 4).

Related studies have reported that L-phenylalanine has a hormonal effect. Short exposure to L-phenylalanine had no effect on *Microcystis aeruginosa* membrane integrity. However, after 24 and 48 h exposure, cells exposed to L-phenylalanine at concentrations between 1.25 and 20 μg/mL showed that L-phenylalanine efficiently inhibited the growth of *M. aeruginosa* by disrupting the cell membrane integrity and inhibiting esterase activity (Wei, 2011). In addition, the metabolites of L-phenylalanine, *trans*-cinnamic acid, and benzoate also had toxic effects in plants, thereby inhibiting the growth of plants. Zhang et al. (2009) reported that benzoate and cinnamic acids could cause the rapid and remarkable downregulation of genes related to the cell cycle, which resulted in the inhibition of cucumber root growth. Moreover, Salvador et al. (2013) found that externally added *trans*-cinnamic acid could be catalyzed into the phenylpropanoid pathway by *trans*-cinnamic acid 4-hydroxylase, thereby increasing the accumulation of *p*-hydroxyphenyl lignin. Meanwhile, as indole-3-acetate oxidase activity increased, related metabolic reactions proceeded to form hardened cell walls, which in turn led to reduced soybean root growth.

The benzoate 4-hydroxylase family was specific to fungi, and detoxification was the major function (Podobnik et al., 2008). Korosec et al. (2014) also proved that tans-cinnamic acid and its derivatives had antifungal activity mainly by inhibiting the activity of benzoate 4-hydroxylase. In this study, the addition of L-phenylalanine resulted in a lower biomass for *G. lucidum*, which may be due to the toxic effects from the metabolic intermediates of L-phenylalanine. The significant increase in the expression of the benzoate 4-hydroxylase gene in the L-phenylalanine metabolism of *G. lucidum* indirectly demonstrated the metabolism of L-phenylalanine to a benzoate.

RT-qPCR analysis showed that the expression level of the related cell wall mannoprotein gene (*pir*, GL18553-R1) was down-regulated by 35.83%. As core components of the fungal cell wall, chitin and glucan need further modification by mannoproteins, so mannoproteins play a vital role in cell wall assembly (Orlean and Menon, 2007; Du et al., 2019). Moreover, the outer layer of the fungal cell wall is mainly formed by mannoprotein and β-glucan (Kollar et al., 1997). Hu et al. (2015) demonstrated that reduced transcription levels of genes encoding mannprotein had a negative impact on cell wall integrity. Thus, the decreasing expression of cell wall mannoprotein gene may lead to the growth inhibition of *G. lucidum* to a certain extent.

In addition, the results of transcriptome showed that the expressions of four genes involved in polysaccharide synthesis were increased compared with the control (Table 2). Researches demonstrated that increasing gene expression of UDP-glucose pyrophosphorylase (UGP), α-phosphoglucomutase (PGM), phosphoglucose isomerase (PGI), and phosphomannose isomerase (PMI) facilitated polysaccharide production (Yang et al., 2013; Li et al., 2015; Xu et al., 2015; Han et al., 2017). Therefore, L-phenylalanine treatment increased the expression level of four genes related to polysaccharide synthesis, which should be one of the reasons for the increase of polysaccharide production.

**TABLE 2:** The expression of genes related to polysaccharide biosynthesis.

**EC no.**	**Gene ID**	**Enzyme name**	**Read count**	
			**L-phenylalanine**	**Control**
5.4.2.2	GL24280-R1	α-phospho- glucomutase (PGM)	73.19	64.93
2.7.7.9	GL25739-R1	UDP-glucose pyrophosphorylase (UGP)	285.53	221.55
5.3.1.9	GL22245-R1	Phosphoglucose isomerase (PGI)	53.11	39.24
5.3.1.8	GL22193-R1	Phosphomannose isomerase (PMI)	31.01	22.60

### The Effect of L-Phenylalanine on the Biosynthesis of the *G. lucidum* Cell Wall

**FIGURE 5:**
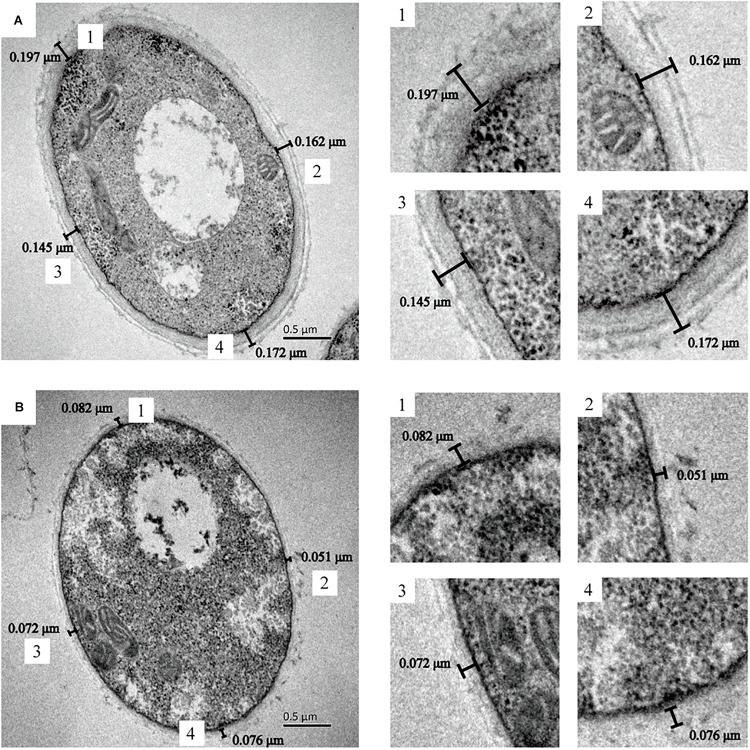
*G. lucidum* cultured in several conditions. TEM pictures obtained after chemical fixation. **(A)** *G. lucidum* was cultivated at 30°C on a rotary shaker at 150 rpm. **(B)** *G. lucidum* was cultivated in a medium containing 0.4 g/L L-phenylalanine, added at 24 h.

The cell wall thickness of *G. lucidum* was observed by TEM (Figure 5). There was a significant difference in mycelial cell wall thickness. The control cell wall (0.169 ± 0.02 μm) was approximately 2.5-fold the thickness of the cells treated with L-phenylamine (0.070 ± 0.01 μm). This result proves that L-phenylalanine has a notable inhibitory effect on the cell wall synthesis of *G. lucidum*. The cell walls of fungi are important to maintain the cell’s intrinsic morphology and the integrity of cells. A network of polysaccharides maintains normal cell metabolism, ion exchange, and osmotic pressure, and also enables the fungal cell to contact the external environment (Bowman and Free, 2006; Tavernier et al., 2018; OuYang et al., 2019). Therefore, a significant decrease in the cell wall’s thickness may be beneficial to the secretion of polysaccharides.

**FIGURE 6:**
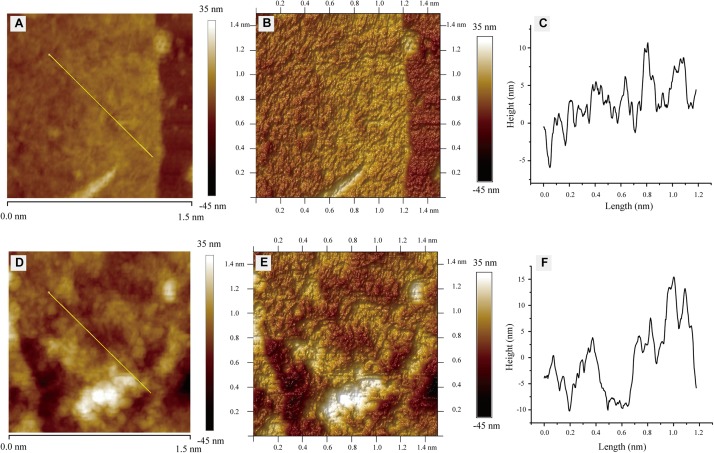
Atomic force microscopy images of *G. lucidum*. **(A)** Two-dimensional image of *G. lucidum*. Straight line is for the line profile analysis. **(B)** Three-dimensional rendering of panel **(A)**. **(C)** A height profile of over 1.2 μm for straight line in panel **(A)**. **(D)** Two-dimensional image of the L-phenylalanine-treated group. A straight line is for line profile analysis. **(E)** Three-dimensional rendering of panel **(D)**. **(F)** The height profile over 1.2 μm for a straight line in panel **(D)**.

In order to further study the effect of L-phenylalanine on the cell wall, the surface morphology of the *G. lucidum* cell wall was observed by AFM (Figure 6). The cell wall surfaces of the control showed an obviously tight network structure (Figure 6A). However, L-phenylalanine treatment had a certain destructive effect on the network structure integrity of the cell wall surface (Figure 6D). In the three-dimensional rendering, the difference in surface fluctuation was more obvious than that of the two-dimensional images (Figures 6B,E). L-phenylalanine treatment resulted in more pores in the network structure of the cell wall surface, which may be related to the fact that the outer layer of the fungal cell wall was mainly composed of cell wall mannoprotein and β-glucan. In section “Comparative Analysis of the *G. lucidum* 5.26 Transcriptomes,” it was also seen that the addition of L-phenylalanine down-regulated the expression of the cell wall mannoprotein gene by 35.83%. The line profile showed surface fluctuations, revealing the significant height differences between the network structure under culture conditions without and with L-phenylalanine (Figures 6C,F). The maximum height of the cell wall surface without L-phenylalanine was 10.69 nm, and the maximum depth was 5.92 nm. The maximum height of the cell wall surface with L-phenylalanine was 15.44 nm, and the maximum depth was 10.21 nm. This result illustrates the negative effect of L-phenylalanine on the cell wall integrity of *G. lucidum*.

**FIGURE 7:**
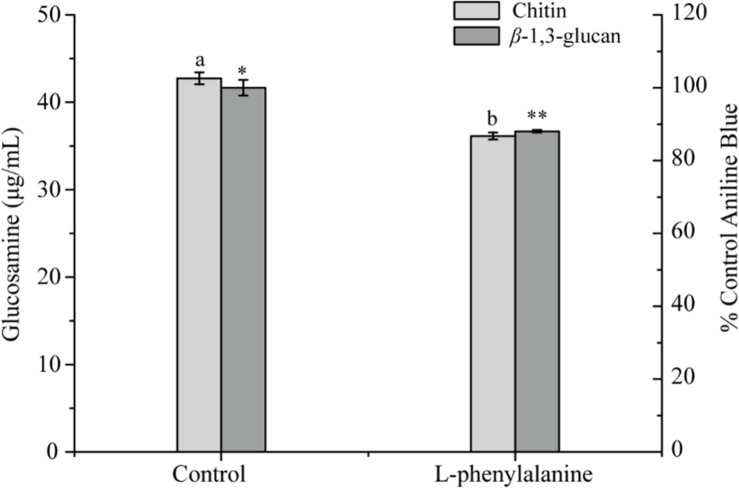
Chitin and β-1,3-glucan concentrations in *G. lucidum* [the different superscript letters (a, b) and symbols (^∗^, ^∗∗^) in each column indicate significant differences at the *P* < 0.05 level].

The contents of chitin and β-1,3-glucan in the cell wall of *G. lucidum* were determined. The results showed that after phenylalanine treatment, the contents of chitin and β-1,3-glucan were significantly reduced by 15.44 and 11.97%, respectively, compared with the control (Figure 7). Chitin has great tensile strength and contributes to the integrity of cell walls (Bowman and Free, 2006). In addition, β-glucan and cell wall mannoprotein, as the main components of the outer layer of the cell wall of fungi, play an important role in the outer structure of the cell wall. It has been suggested that a decrease in chitin and β-1,3-glucan content would result in great damage to the integrity of the cell wall (Kollar et al., 1997). The results were consistent with those observed by TEM and AFM.

Mannoproteins plays a crucial role in cell wall assembly and can further modify chitin and glucan. Moreover, mannoproteins are also directly related to cell wall porosity. A decrease in mannoproteins will increase the porosity of the cell wall (Zlotnik et al., 1984; De Nobel et al., 1989). Therefore, a decrease in the *G. lucidum* cell wall thickness and an increase of the cell wall pores may be mainly caused by the down-regulated expression level of the cell wall mannoprotein gene. In addition, the cell wall probably acts as a molecular sieve, and its porosity directly determines macromolecules’ exportation abilities (De Nobel and Barnett, 1991). It has been certified that yeast macromolecule secretion is linked to high cell wall porosity (Boivin et al., 1998). In Figure 2A, after L-phenylalanine treatment, the mycelium Q_EPS_ increased 1.71-fold compared with the control. Therefore, a decrease in cell wall thickness and an increase in cell wall pores probably facilitated the secretion rate of EPS, thereby leading to increased EPS production.

## Conclusion

The EPS production of *G. lucidum* increasing by 62.50% by adding 0.4 g/L L-phenylalanine after a 24 h culture. The possible metabolic pathway of L-phenylalanine in *G. lucidum* was deduced. Additionally, L-phenylalanine and its metabolic intermediates promoted the production of IPS and had a significant inhibitory effect on the cell wall synthesis of *G. lucidum*. In conclusion, considering the wide production range and low price of L-phenylalanine, it is economically feasible to increase the EPS production of *G. lucidum* by L-phenylalanine.

## Data Availability Statement

The datasets analyzed in this manuscript are not publicly available. Requests to access the datasets should be directed to bioding@163.com.

## Author Contributions

ZM and ZD designed the experiments. ZM, MX, and QW performed the experiments. ZM, FW, HZ, ZG, YL, GS, and ZD conceived the project, analyzed the data, and wrote the manuscript.

## Conflict of Interest

HZ was employed by Jiangsu Alphay Biological Technology Co., Ltd. The remaining authors declare that the research was conducted in the absence of any commercial or financial relationships that could be construed as a potential conflict of interest.

## Abbreviations

AFM: atomic force microscope
DW: dry weight
EPS: extracellular polysaccharide
IPS: intracellular polysaccharide
KAAS: KEGG automatic annotation server
TEM: transmission electronic microscopy
